# Alterations in the diversity and composition of the fecal microbiota of domestic yaks (*Bos grunniens*) with pasture alteration-induced diarrhea

**DOI:** 10.1186/s12917-024-04196-4

**Published:** 2024-08-09

**Authors:** Runbo Luo, DanWu, Zhengzhong Luo, Yupeng Li, Yanan Zhong, Kexin Li, Zhanchun Bai, Gongga, Suolangsizhu

**Affiliations:** 1 College of Animal Science, Xizang Agricultural and Animal Husbandry University, Linzhi, 860000 China; 2 College of Veterinary Medicine, Sichuan Agricultural University, Chengdu, 611130 China

**Keywords:** Yak (*Bos grunniens)*, Diarrhea, Pasture alteration, Fecal microbiota, 16S RNA amplicon sequencing

## Abstract

Diarrhea is a common issue in domestic yaks (*Bos grunniens*) that can occur with pasture alterations and significantly impacts growth performance. Previous research has examined the microbiota of diarrhetic yaks; however, the structural changes in gut bacterial community and microbial interactions in yaks with grassland alteration-induced diarrhea remain poorly understood. To explore variations in gut microbiota homeostasis among yaks suffering from diarrhea, fecal microbiota diversity and composition were analyzed using 16 S rRNA amplicon sequencing. Gut fecal microbiota diversity was lower in diarrhetic yaks than in non-diarrhetic yaks. Furthermore, the bacterial community composition (including that of Proteobacteria and Actinobacteria) in the feces of diarrhetic yaks displayed significant alterations. Co-occurrence network analysis further underscored the compromised intestinal flora stability in yaks with diarrhea relative to that in non-diarrhetic yaks. Interestingly, the abundance of beneficial bacteria, such as *Lachnospiraceae_AC2044_group* and *Lachnospiraceae_NK4A136_group*, were decreased in yaks with diarrhea, and the reductions were negatively correlated with the fecal water content. Collectively, these findings indicate that diminished microbial stability and increased abundance of certain bacteria in the gut may contribute to diarrhea occurrence in yaks.

## Introduction

The domestic yak (*Bos grunniens*) is a distinct livestock breed found in the Asian highlands, and it is significantly correlated to the lives of local residents. Yaks are present in many countries, including China, India, Bangladesh, Pakistan, and Tajikistan; however, the main yak breeding regions are predominantly situated in the Qinghai-Tibet Plateau [1]. Yaks profoundly influence the lifestyle of individuals on the Qinghai-Tibet Plateau through their roles in transportation, fuel provision, milk production, and meat production [2]. Unlike the wild yak (*Bos mutus*), domestic yaks are primarily raised using intensive grazing techniques and are regularly confined to alternative grazing systems throughout the year [3]. However, domestic yaks suffer from pasture challenges and climatic changes during the transition from summer to winter [4, 5]. Alterations in pastures can affect certain behavioral characteristics of yaks, such as grazing time, rumination time, walking distance, idling periods, and drinking frequency [5]. In addition, domestic yaks are challenged by a high disease incidence, particularly, diarrhea and pneumonia during the transition period of pasture alteration or feed methods [6–8]. However, the factors that contribute to these health issues in *B. grunniens* remain unclear.

Emerging research suggests that the unique genetic architecture of yaks allows them to adapt to the environmental challenges of high altitudes, including extreme cold, food scarcity, and low oxygen levels [9, 10]. Recently, the gut microbiome has been regarded as the “second genome” of the animal body and is closely associated with health and environmental adaptation [11, 12]. The gut microbiota dynamics in yaks at high altitudes undergo significant changes in response to seasonal variations and transitions between grasslands [13, 14]. However, gut microbiota dysbiosis is an important contributor to many diseases, including metabolic disease, diarrhea, and infectious diseases [12, 15]. Based on 16 S rRNA high-throughput sequencing, a previous study reported that diverse microbial communities in yak feces are associated with weaning diarrhea development [16]. Although the fecal microbiota of diarrhetic yaks have been reported [7], structural changes in the bacterial community and microbial interactions in the yak gut with grassland alteration-induced diarrhea remain poorly understood.

To investigate the relationship between the gut microbial community and diarrhea in yaks induced by pasture alterations, we analyzed and compared the diversity and composition of the fecal microbiota in non-diarrhetic and diarrhetic yaks following the transition from summer to winter pastures. Microbial interactions and their associations with fecal characteristics were examined based on a correlation analysis. Our data provide insights into diarrhea pathogenesis in yaks and offer a scientific basis for improving yak health through the regulation of gut microbiota homeostasis.

## Materials and methods

### Animals and sample collection

The animals used in this study were obtained from a herd of 500 yaks, which were raised at a yak farm located in the Aba Tibetan Autonomous Prefecture, Sichuan Province. Yaks graze year-round, and the grassland rotates twice annually. Summer and winter pastures were used from June to September and from October to May, respectively. Each yak was fitted with ear tags on both ears. Throughout the experimental period, the health of the yak herd was monitored by veterinarians, and yaks only experiencing diarrhea were separated. Diarrhea was diagnosed according to the criteria established in a previous report [17]. Fecal samples were collected from both diarrhetic (*n* = 14) and non-diarrhetic (*n* = 12) yaks of similar weight from October to December 2023. The body weights of non-diarrhetic and diarrhetic yaks were 135.5 ± 21.7 kg and 127.3 ± 20.5 kg (mean ± SD), respectively.

### Evaluation of fecal characteristics

**Fig. 1:**
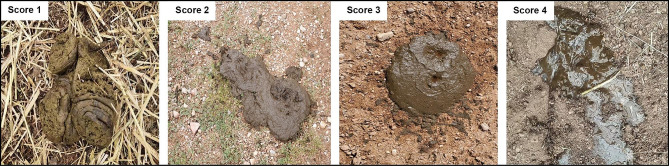
Yak fecal score chart. Scores range from 1 to 4, with 1 indicating very firm feces and 4 indicating watery diarrhea

Fecal consistency was assessed using the Fecal Scoring Guide (Alltech^®^) as follows: score 1, dry and hard; score 2, soft and formed into a paste; score 3, mix of liquids with some solids; and score 4, liquid and watery (Fig. 1). Scores 1 and 2 fecal samples were obtained from non-diarrhetic yaks, while scores 3 and 4 were collected from diarrhetic yaks. A 500 g fecal sample was dehydrated in a forced-air oven (GZX-9140MBE, Shanghai BoXun Medical Biological Instruments Co., Ltd. Shanghai, China) at 65 °C for 48 h to determine the dry weight, which was subsequently utilized to calculate the fecal water content.

### 16S RNA amplicon sequencing analysis of feces

Total DNA was extracted from 26 yak fecal samples using a Stool DNA Kit (Tiangen Biotech Co., Ltd., Beijing, China). The quality and quantity of the extracted DNA were assessed via electrophoresis on a 1.8% agarose gel, and the DNA concentration and purity were determined using a NanoDrop 2000 UV-Vis spectrophotometer (Thermo Scientific, Wilmington, DE, USA) [18]. In this study, the DNA concentration was determined to be 18.95 ± 4.68 ng/µL, meeting the necessary criteria for amplicon preparation and SMRTbell library construction. The full-length 16 S rRNA gene was amplified using primer pairs 27 F: AGRGTTTGATYNTGGCTCAG and 1492R: TASGGHTACCTTGTTASGACTT [19]. PCR amplicons were purified using VAHTSTM DNA Clean Beads (Vazyme, Nanjing, China) and quantified using a Qubit dsDNA HS Assay Kit and Qubit 3.0 Fluorometer (Invitrogen, Thermo Fisher Scientific, Oregon, USA). After individual quantification, amplicons were combined in equal proportions. SMRTbell libraries were constructed from amplified DNA using the SMRTbell Express Template Prep Kit 2.0, according to the manufacturer’s protocol (Pacific Biosciences). The purified SMRTbell libraries from the pooled and barcoded samples were sequenced on a PacBio Sequel II platform (Beijing Biomarker Technologies Co., Ltd., Beijing, China) using the Sequel II Binding Kit 2.0.

### Data processing

Raw reads from 16 S rRNA sequencing were processed using SMRT Link software (v8.0) for filtering and demultiplexing. Specific parameters (minPasses ≥ 5 and minPredictedAccuracy ≥ 0.9) were applied to generate circular consensus sequencing reads. Subsequently, sequences were assigned to their respective samples based on barcodes using Lima software (v1.7). Filtering steps, including primer identification, quality control, and length range filtering (1200–1650 bp), were performed to remove unwanted reads. Chimeric sequences were detected and eliminated using the UCHIME algorithm (v8.1) to obtain clean reads. Operational taxonomic units (OTUs) were defined using USEARCH (v10.0) based on sequences with > 97% similarity. Taxonomic annotation of the OTUs was performed using the naïve Bayes classifier in QIIME2 utilizing the SILVA database (release 138.1) with a confidence threshold of 70% [20].

### Statistical analysis

Alpha diversity analysis was conducted to assess the complexity of species diversity in each sample using the *Vegan* package (v2.6) in R. Beta diversity analysis was performed using principal coordinate analysis (PCoA) to evaluate the complexity of species diversity among samples. Comparisons of bacterial abundance and diversity between the feces of non-diarrhetic and diarrhetic yaks were performed using the Mann–Whitney U test. Taxa differential abundances were evaluated using linear discriminant analysis effect size (LEfSe). The phyla co-occurrence network was analyzed using the *igraph* R package (v2.0), with network generation and topological analysis executed using Gephi software. Spearman’s rank correlation analyses were conducted to assess the relationship between key microbes and fecal characteristics. Graphical representations were created using GraphPad Prism (v.10) and the *ggplot2* R package (v3.5).

## Results

### Changes in the characteristics of diarrhetic yak feces

**Fig. 2:**
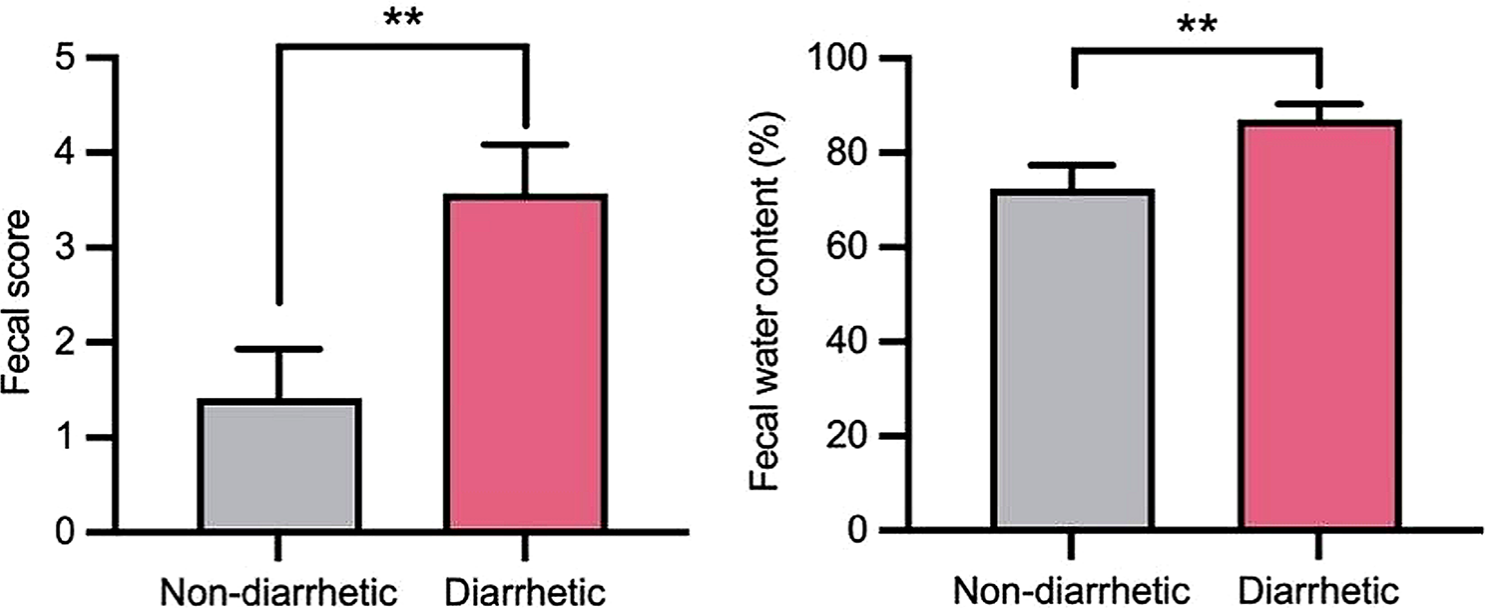
Comparison of fecal scores and fecal water content between non-diarrhetic yaks (*n* = 12) and diarrhetic yaks (*n* = 14). ^**^ Significant difference at the *p* < 0.01 level

During the study, the diarrhea incidence in yaks was 4.2% when the grazing area transitioned from summer to winter pasture. Evaluation of the fecal characteristics revealed that the fecal score and water content of diarrhetic yaks were significantly higher (*p* < 0.01) than those of non-diarrhetic yaks (Fig. 2).

### Changes in gut microbiota diversity and composition of diarrhetic yak feces

**Fig. 3:**
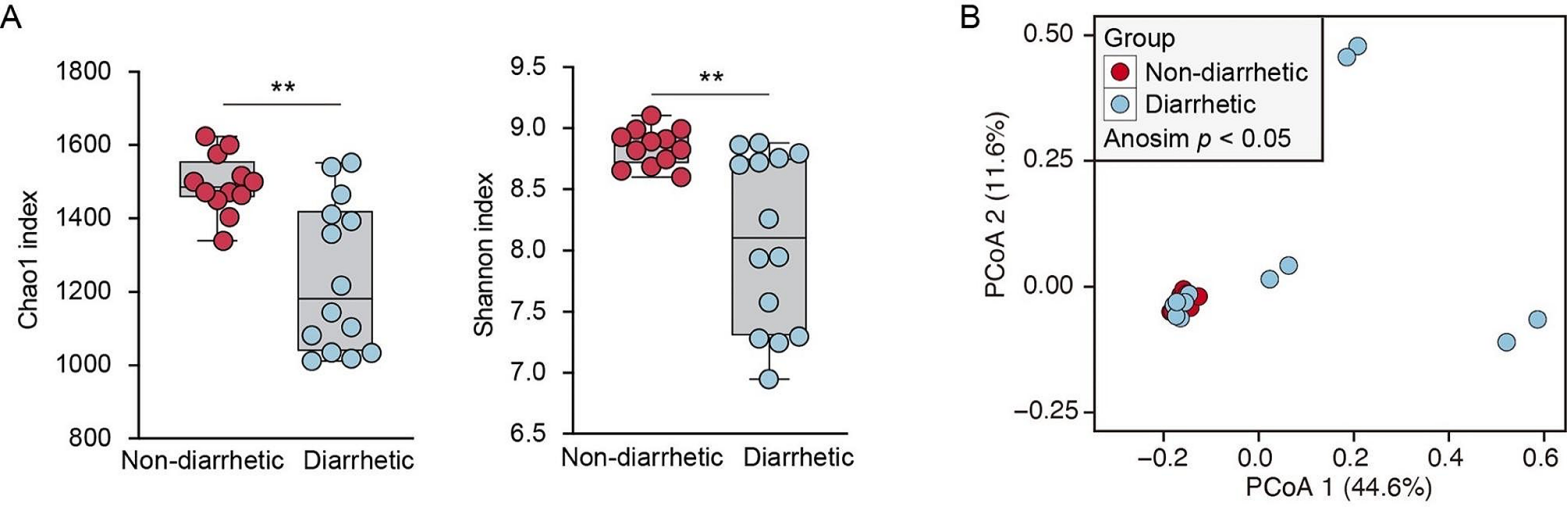
Comparison of fecal microbiota diversity between non-diarrhetic and diarrhetic yaks. (**A**) Alpha diversity, including Chao1 and Shannon indexes. (**B**) Beta diversity. ^**^ Significant difference at the *p* < 0.01 level

The results of the alpha diversity analysis showed that the Chao1 and Shannon indices of the gut microbiota in diarrhetic yaks were significantly lower (*p* < 0.01) than those in non-diarrhetic yaks, indicating a noticeable alteration in the diversity and richness of the gut microbiota in yaks with diarrhea (Fig. 3). Based on the PCoA analysis, significant differences in microbial community structure between diarrhetic yaks and non-diarrhetic yaks were observed (ANOVA, *p* < 0.05).

**Fig. 4:**
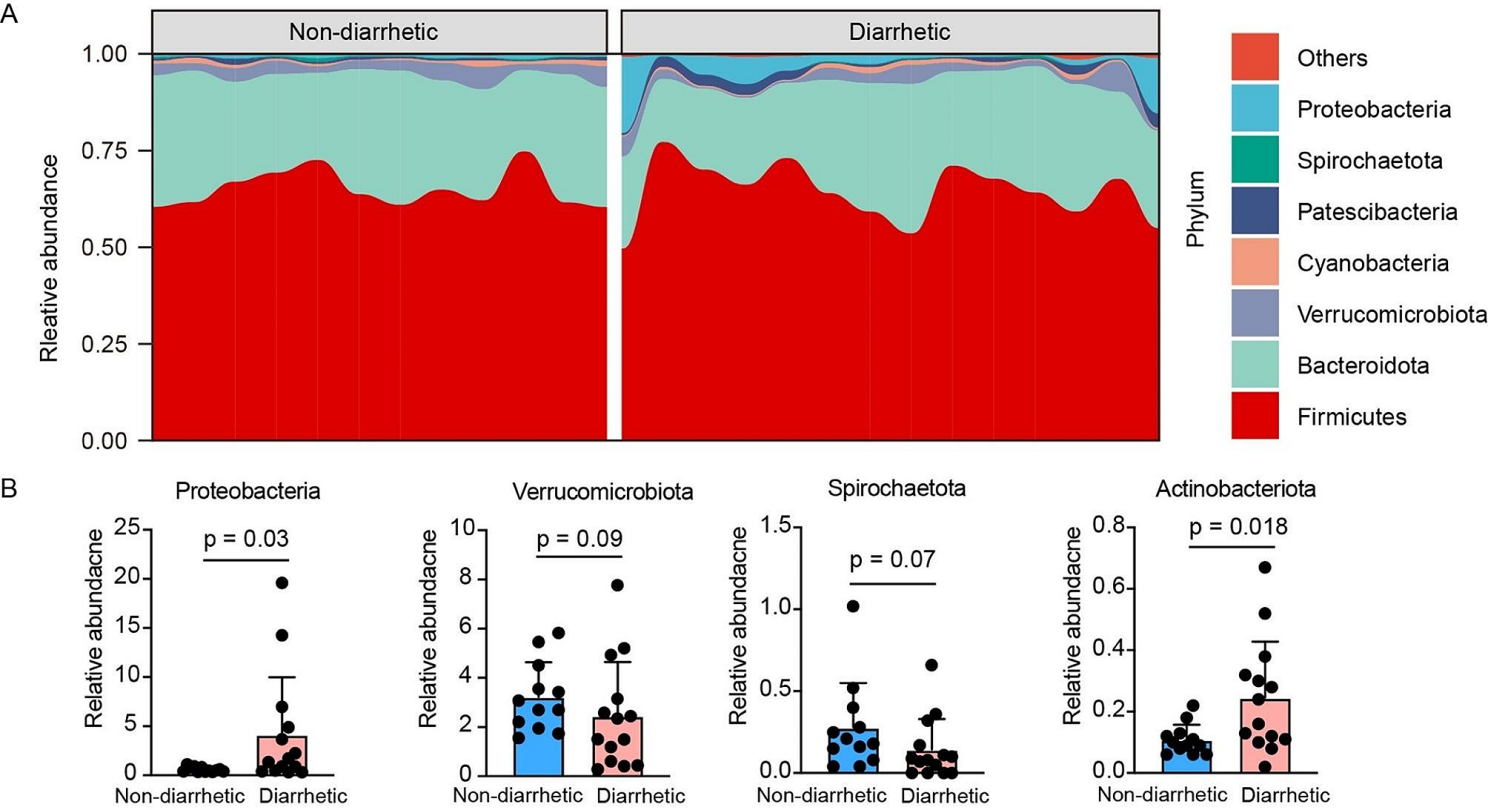
Composition of fecal microbiota communities in domestic yaks. (**A**) Staked plot showing the relative bacterial abundance (%) at the phylum level between diarrhetic and non-diarrhetic yaks. (**B**) Bar plots displaying differential phylum taxa (relative abundance > 0.1% only) between diarrhetic and non-diarrhetic yaks

Analysis of the gut microbiota composition in yak feces revealed that Firmicutes, Bacteroidetes, Verrucomicrobiota, and Proteobacteria were the dominant bacterial phyla (Fig. 4A). Notably, the relative abundance of Proteobacteria in the feces of diarrhetic yaks was significantly higher (*p* = 0.03) than in that of non-diarrhetic yaks (Fig. 4B). Furthermore, the relative abundance of Verrucomicrobiota and Spirochaetota in the feces of diarrhetic yaks was lower (*p* < 0.1) than in that of non-diarrhetic yaks. Actinobacteria relative abundance in yaks with diarrhea was markedly higher (*p* = 0.018) than that in non-diarrhetic yaks.

**Fig. 5:**
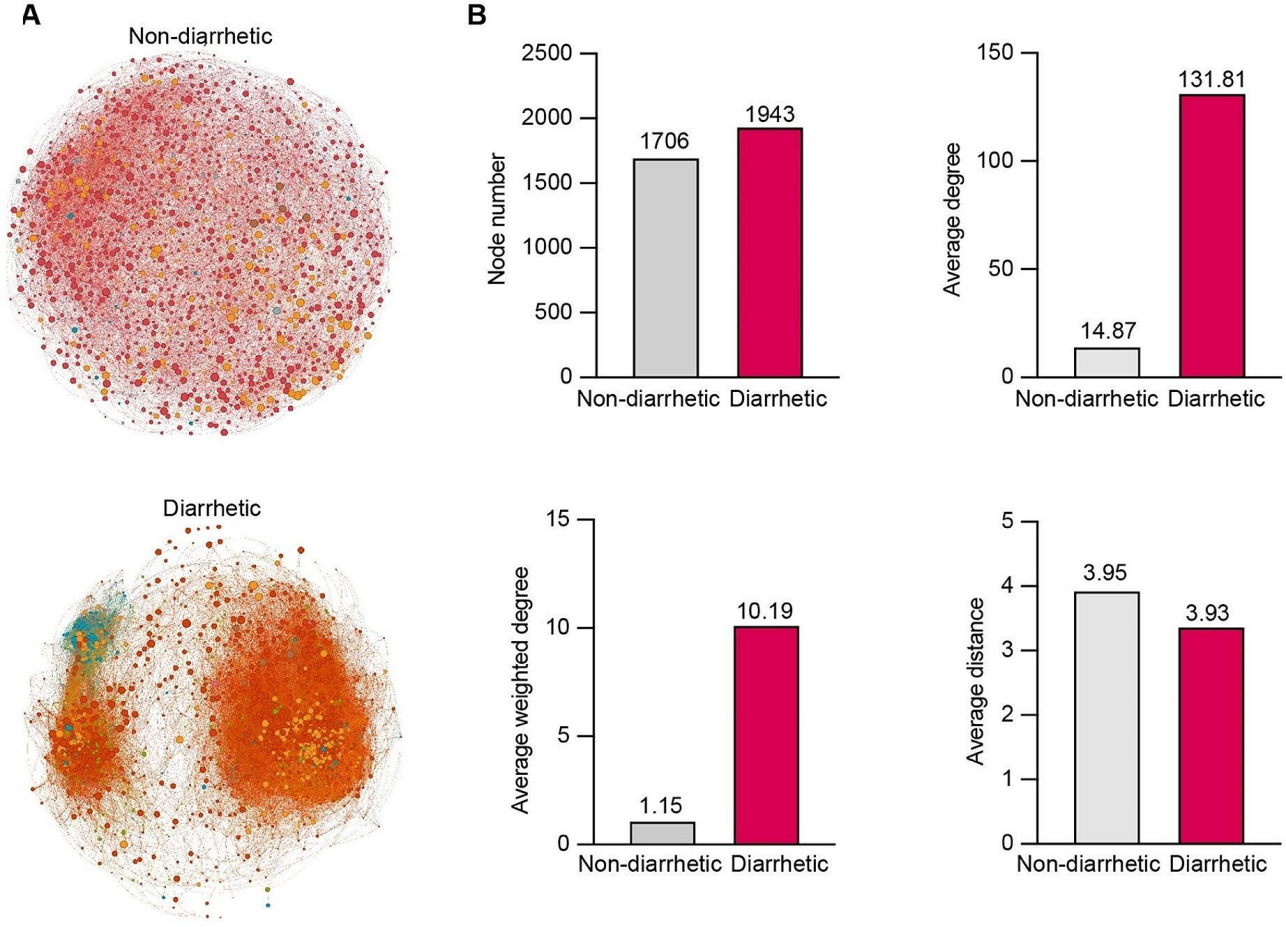
Co-occurrence network (**A**) and network topology (**B**) of bacterial communities between diarrhetic and non-diarrhetic yaks at the phylum level. The node size corresponds to phylum taxa relative abundance. Node color indicates the different phylum taxa

To investigate variations in gut microbial interactions, we conducted a co-occurrence network analysis at the phylum level. Bacterial community interactions differed between diarrhetic and non-diarrhetic yaks (Fig. 5A). The number of nodes in the co-occurrence network was higher in diarrhetic yak feces (1.14-fold) than in non-diarrhetic yak feces (Fig. 5B). The average and weighted degrees of bacterial communities in diarrhetic yaks were higher than those in non-diarrhetic yaks (8.86-fold). In addition, the average network distances were similar in bacterial communities between the non-diarrhetic and diarrheal yaks (3.95 and 3.93, respectively).

### Alterations in the gut microbiota of diarrhetic yak feces

**Fig. 6:**
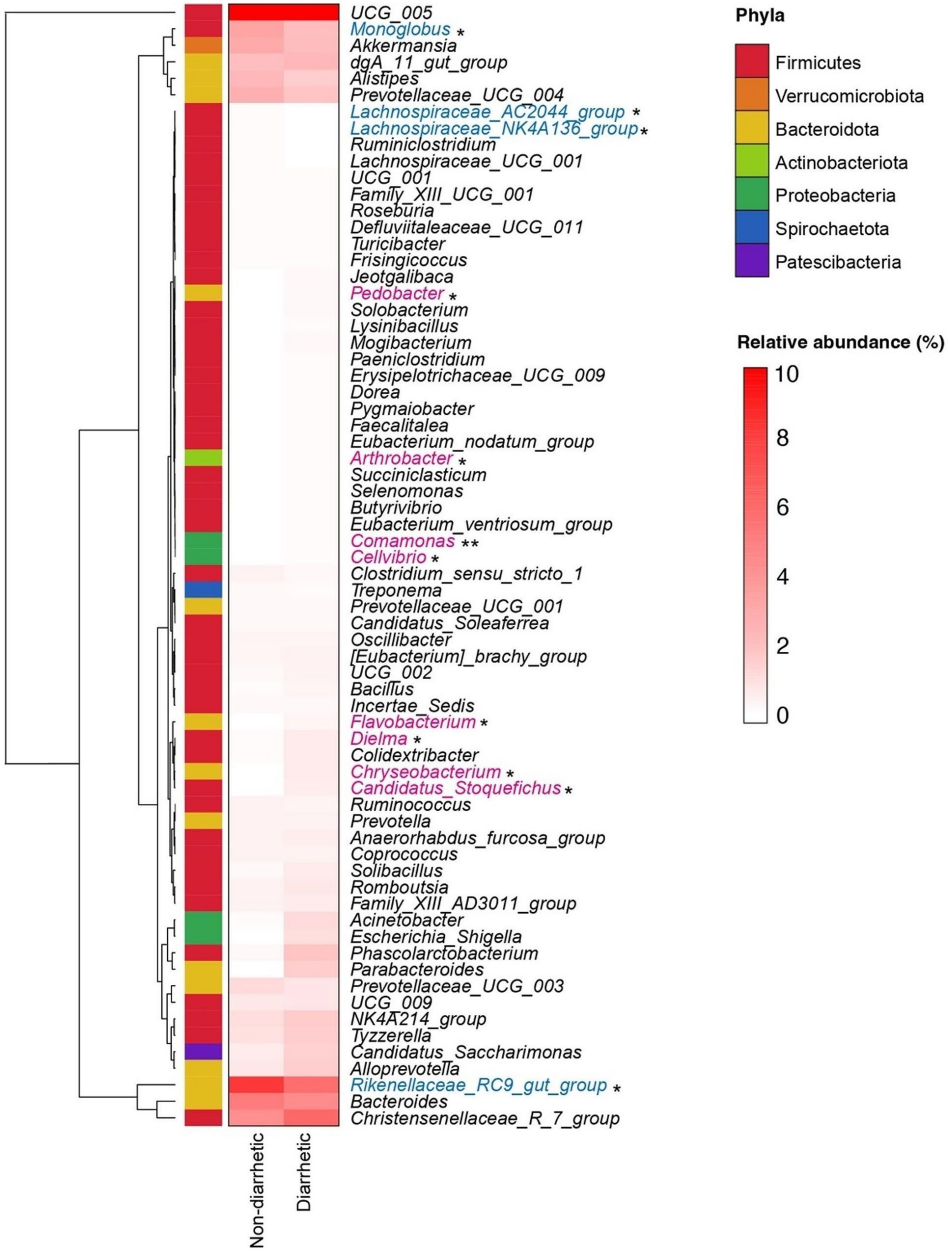
Heatmap displaying the relative abundance of genera in yak feces (only abundances > 0.1% are included). Genera marked in blue indicate a lower relative abundance in diarrhetic yaks than in non-diarrhetic yaks, while genera in red indicate a higher relative abundance in diarrhetic yaks than in diarrhetic yaks. ^*^ and ^**^ Significant differences at the *p* < 0.05 and *p* < 0.01 level, respectively

**Fig. 7:**
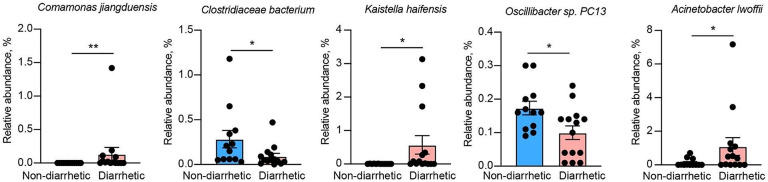
Relative abundances of differential bacteria between diarrhetic and non-diarrhetic yaks. ^*^ and ^**^ Significant differences at the *p* < 0.05 and *p* < 0.01 level, respectively

At the genus level, *UCG_005*, *Rikenellaceae_RC9_gut_group*, *Bacteroides*, *Christensenellaceae_R_7_group*, *Monoglobus*, and *Akkermansia* were the six most abundant genera in diarrhetic yak feces (Fig. 6). The relative abundances of *Lachnospiraceae_AC2044_group*, *Lachnospiraceae_NK4A136_group*, *Monoglobus*, and *Rikenellaceae_RC9_gut_group* were significantly lower (*p* < 0.05) in yaks with diarrhea than in non-diarrhetic yaks. The relative abundances of *Comamonas*, *Dielma*, *Chryseobacterium*, *Flavobacterium*, *Acinetobacter*, and *Candidatus_Stoquefichus* were significantly higher (*p* < 0.05) in diarrhetic yaks than in non-diarrhetic yaks. The abundance of *Comamonas jiangduensis* (belonging to the *Comamonas* genus) was significantly higher (*p* < 0.01) in diarrhetic yaks than in non-diarrhetic yaks (Fig. 7). In addition, the relative abundances of *Albibacterium bauzanense* and *Kaistella haifensis* were significantly higher (*p* < 0.05) and the relative abundances of *Clostridiaceae bacterium* and *Oscillibacter sp.PC13* were significantly lower (*p* < 0.05) in diarrhetic yaks than in non-diarrhetic yaks. Moreover, the relative abundance of *Acinetobacter lwoffii* (belonging to the *Acinetobacter* genus) was higher (*p* = 0.039) in diarrhetic yaks than in non-diarrhetic yaks.

**Fig. 8:**
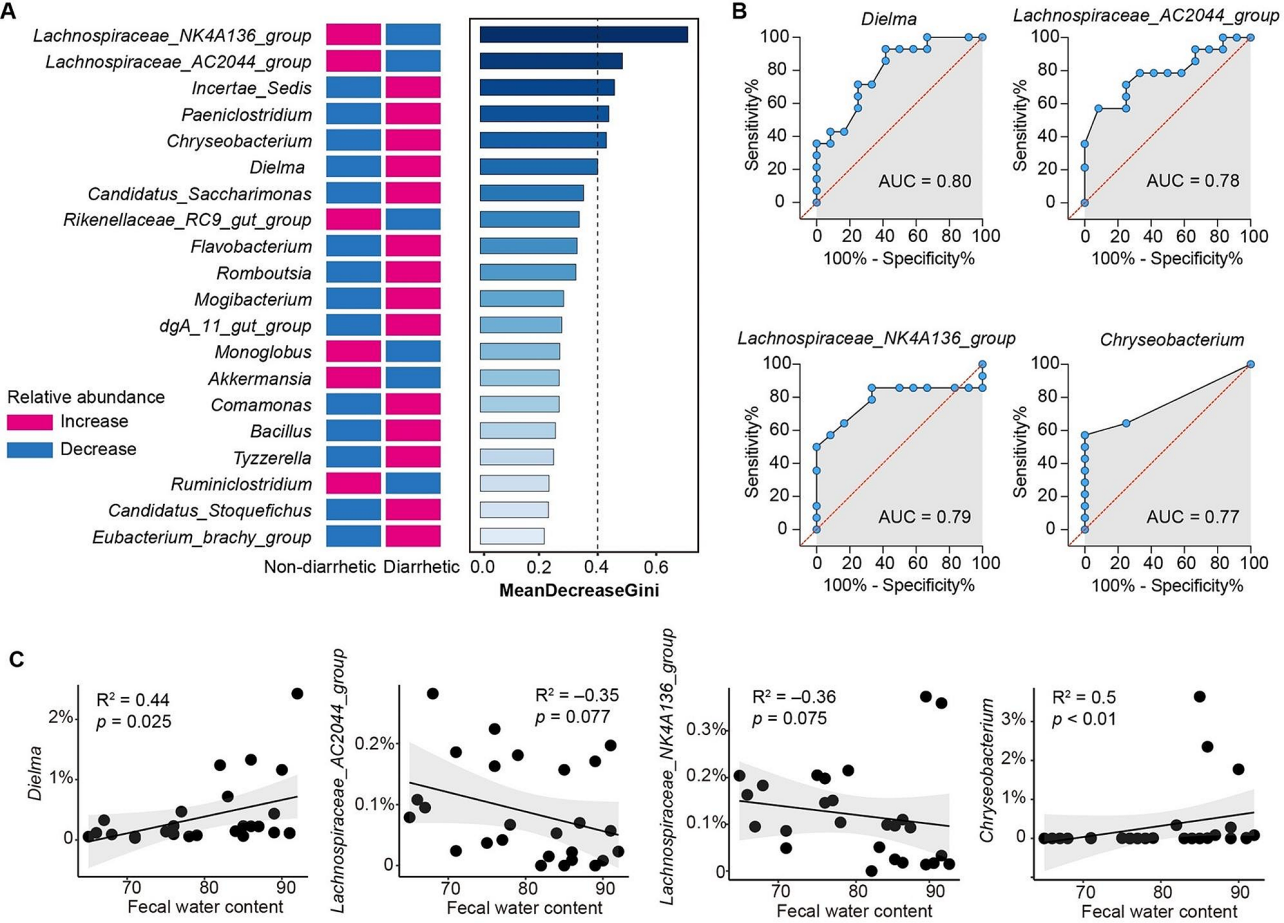
Determination of key genera in diarrhetic yak feces. (**A**) Bar plot showing the top 20 genera ranked by mean decrease Gini in random forest analysis. (**B**) Four differential genera were identified based on random forest analysis and a Wilcoxon rank sum test. Differential genera were evaluated using receiver operator characteristic curve analysis. (**C**) Spearman’s rank correlation between fecal water content and relative abundance of key genera in yak feces

To screen for key genera involved in the development of diarrhea, a random forest analysis was performed. *Lachnospiraceae_AC2044_group*, *Lachnospiraceae_NK4A136_group*, *Chryseobacterium*, *Paeniclostridium*, and *Incertae_Sedis* were important contributors to diarrhea incidence in yaks (Fig. 8A). Based on our Wilcoxon rank sum test and receiver operator characteristic curve analysis, *Lachnospiraceae_AC2044_group* (AUC = 0.78), *Lachnospiraceae_NK4A136_group* (AUC = 0.79), *Dielma* (AUC = 0.80), and *Chryseobacterium* (AUC = 0.77) were identified as the key genera in diarrhetic yaks (Fig. 8B). In addition, increases in the relative abundances of *Dielma* and *Chryseobacterium* were strongly positively correlated (*r* = 0.44, *p* = 0.025 and *r* = 0.5, *p* < 0.01, respectively) with the fecal water content (Fig. 8C). The decrease in relative abundances of *Lachnospiraceae_AC2044_group* and *Lachnospiraceae_NK4A136_group* were negatively correlated with fecal water content (*r* = -0.35, *p* = 0.077 and *r* = -0.36, *p* = 0.075, respectively).

## Discussion

The gut microbiota plays an important role in the high-altitude adaptability and health of livestock on the Qinghai-Tibet Plateau [21]. At high altitudes, bacterial community diversity in the large intestines of non-diarrhetic yaks is relatively stable [22]. However, yaks usually suffer from diseases, particularly during winter [5, 23]. A previous study showed that the diversity of the gut microbiota community was altered in yaks with diseases, such as diarrhea and parasitization [7, 24]. In this study, we found that alpha diversity indices in the fecal microbiota, such as Chao1 and Shannon, were lower in yaks with diarrheal events. Similar to our findings, Wu et al. [25] and Han et al. [26] suggested that the species richness and evenness of fecal microbiota was lower in diarrhetic yaks. In feedlot cattle, the Chao1 and Shannon indices of bacterial diversity in fecal samples were determined in cases of hemorrhagic diarrhea [27]. In addition, gut microbiota composition differed markedly between non-diarrhetic and diarrhetic yaks [7, 25], which is consistent with the results of our study. Compared with the diversity of the gut microbiota community in yaks, the diversity in dairy cows did not significantly differ [28, 29]. Although seasonal diets can affect microbial community diversity [30], alterations in gut microbiota diversity may contribute to reduced adaptive capacity in yaks and lead to disease onset.

Microbiota in the gastrointestinal tract of cattle, including yaks, dairy cows, and beef cattle have similar compositions [12, 31]. Major phyla present in the gastrointestinal tract include Firmicutes and Bacteroidetes. Dai et al. [32] reported that the relative abundances of Firmicutes and Bacteroidetes were higher in the feces of yaks, and the Firmicutes to Bacteroidetes ratio did not differ between wild and domestic yaks. In this study, we observed that Firmicutes, Bacteroidetes, Verrucomicrobiota, and Cyanobacteria were the major bacterial taxa present in yak feces. Meanwhile, the relative abundances of Proteobacteria and Actinobacteria in diarrhetic yak feces were higher than those in non-diarrhetic yak feces. Compared with other core microbiota, Proteobacteria is regarded as a microbial signature of gut dysbiosis that is closely associated with the development of inflammatory bowel disease [33, 34]. Braun et al. [35] reported that Proteobacteria relative abundance is higher in patients with infectious diarrhea than in non-diarrhetic individuals. Sun et al. [36] and Wu et al. [25] showed that a high relative abundance of Proteobacteria was associated with the development of diarrhea in livestock. Actinobacteria is a commonly observed phylum in the gut. As reported by Li et al. [37], the relative abundance of Actinobacteria is higher in neonatal calves experiencing episodes of diarrhea than that in non-diarrhetic calves. In addition, the relative abundance of Verrucomicrobiota decreased in yaks with grassland alteration-induced diarrhea. Verrucomicrobiota is a phylum of gram-negative bacteria that participates in the regulation of energy metabolism, as reported in a previous study [38]. Recent studies have indicated that Akkermansia, a member of the phylum Verrucomicrobiota, plays a critical role in maintaining intestinal health by modulating the production of short-chain fatty acids and inflammatory factors [39]. The genus *Akkermansia* exhibited a high abundance in the bacterial composition of both non-diarrhetic and diarrhetic yaks in this study, although the two groups did not significantly differ, which is consistent with previous reports [25].

Our study found that the relative abundances of *Lachnospiraceae_AC2044_group* and *Lachnospiraceae_NK4A136_group* were lower in diarrhetic yaks than in non-diarrhetic yaks and negatively correlated with the fecal water content. *The Lachnospiraceae_NK4A136_group* is a potential probiotic that produces large amounts of butyrate to maintain biological functions associated with health [40]. *Lachnospiraceae_NK4A136_group* abundance decreases during colitis, whereas dietary nutrients increase the abundance of this genus, leading to a reduction in colitis severity [41, 42]. The *Lachnospiraceae_AC2044_group*, similar to other *Lachnospiraceae* members, has been associated with the secretion of the glucagon-like peptide 1 in the intestine, which plays a role in regulating glucose homeostasis [43]. Dai et al. [32] found that changes in *Lachnospiraceae_AC2044_group* relative abundance could potentially affect yak growth performance and nutrient digestibility. Additionally, the relative abundances of *Chryseobacterium* and *Dielmma* were higher in yaks with diarrhea than those in non-diarrhetic yaks. *Chryseobacterium* is a genus of gram-negative bacteria known for its role in infectious diseases, including pneumonia and myositis [44]. Previous studies have indicated that *Chryseobacterium* acts as an environmental pathogen leading to diarrhea [45, 46]. Therefore, a reduction in beneficial bacteria and an increase in harmful bacteria are potential contributing factors to the incidence of diarrhea in yaks. However, the causal relationship between these factors and diarrhea occurrence requires further investigation.

## Conclusions

Diarrhea often occurs during the transition from summer to winter pastures. Our study revealed significant alterations in the diversity of the gut microbiota community and topology of microbial interactions in yaks experiencing diarrhea. Furthermore, a notable reduction in the relative abundance of beneficial bacteria was observed in the gut of diarrhetic yaks, which was correlated with an increase in fecal water content. This study contributes to our understanding of the pathogenic mechanisms underlying diarrhea occurrence in yaks and offers insights into microbial composition manipulation through nutritional strategies. Further research in this area facilitates the development of targeted interventions of the gut microbiota to improve overall livestock health and well-being.

## Data Availability

Sequencing data were deposited in the NCBI Sequence Read Archive (SRA) database under the study accession code PRJNA1094449.

## Abbreviations

PCoA: Principal coordinate analysis
LEfSe: Linear discriminant analysis effect size
AUC: Area under the curve
OTU: Operational taxonomic unit
